# Integrated Weighted Gene Co-expression Network Analysis with an Application to Chronic Fatigue Syndrome

**DOI:** 10.1186/1752-0509-2-95

**Published:** 2008-11-06

**Authors:** Angela P Presson, Eric M Sobel, Jeanette C Papp, Charlyn J Suarez, Toni Whistler, Mangalathu S Rajeevan, Suzanne D Vernon, Steve Horvath

**Affiliations:** 1 Biostatistics, University of California, Los Angeles, CA, USA; 2 Pediatrics, University of California, Los Angeles, CA, USA; 3 Human Genetics, University of California, Los Angeles, CA, USA; 4 Division of Viral and Rickettsial Diseases, National Center for Zoonotic, Vector-Borne and Enteric Diseases, Centers for Disease Control and Prevention, Atlanta, GA, USA; 5 Chronic Fatigue and Immune Dysfunction Syndrome (CFIDS), PO Box 220398, Charlotte, NC, USA

## Abstract

**Background:**

Systems biologic approaches such as Weighted Gene Co-expression Network Analysis (WGCNA) can effectively integrate gene expression and trait data to identify pathways and candidate biomarkers. Here we show that the additional inclusion of genetic marker data allows one to characterize network relationships as causal or reactive in a chronic fatigue syndrome (CFS) data set.

**Results:**

We combine WGCNA with genetic marker data to identify a disease-related pathway and its causal drivers, an analysis which we refer to as "Integrated WGCNA" or IWGCNA. Specifically, we present the following IWGCNA approach: 1) construct a co-expression network, 2) identify trait-related modules within the network, 3) use a trait-related genetic marker to prioritize genes within the module, 4) apply an integrated gene screening strategy to identify candidate genes and 5) carry out causality testing to verify and/or prioritize results. By applying this strategy to a CFS data set consisting of microarray, SNP and clinical trait data, we identify a module of 299 highly correlated genes that is associated with CFS severity. Our integrated gene screening strategy results in 20 candidate genes. We show that our approach yields biologically interesting genes that function in the same pathway and are causal drivers for their parent module. We use a separate data set to replicate findings and use Ingenuity Pathways Analysis software to functionally annotate the candidate gene pathways.

**Conclusion:**

We show how WGCNA can be combined with genetic marker data to identify disease-related pathways and the causal drivers within them. The systems genetics approach described here can easily be used to generate testable genetic hypotheses in other complex disease studies.

## Background

Network approaches provide a means to bridge the gap from individual genes to complex traits. Methods for inferring gene interactions from expression data have been an active area of systems biology research [1-6]. Gene set enrichment analysis (GSEA) determines whether an *a priori *defined set of genes shows statistically significant differences between two biological states [7]. In contrast, Weighted Gene Co-expression Network Analysis (WGCNA) constructs gene sets (modules) from the observed gene expression data. These modules are then related to gene ontology information to study their biological plausibility and to eliminate spurious modules due to technical artifacts. Although WGCNA shares the philosophy of GSEA by focusing on gene sets as opposed to individual genes, it does not make use of *a priori *defined gene sets [8]. Instead, modules are constructed from the expression data by using unsupervised clustering. Although it is advisable to relate the resulting modules to gene ontology information for assessing their biological plausibility, it is not required. WGCNA has been successfully applied to identify brain cancer genes [9], to characterize genes related to body weight in mice [10,11], and to study atherosclerosis [12].

WGCNA alleviates the multiple testing problem inherent in microarray data analysis. Instead of relating thousands of genes to the trait, WGCNA relates only a few modules. Because the modules may correspond to biological pathways, focusing the analysis on modules amounts to a biologically motivated data reduction scheme. If genetic marker data are available, one can use genetic marker-based causality tests to identify the genetic drivers underlying the modules of interest. The concept of conducting a causality analysis based on genetic marker data has been explored by several authors [13-22]. We refer to a weighted gene co-expression network analysis that uses genetic markers in causality testing as "Marker Integrated WGCNA" or simply as "IWGCNA".

IWGCNA relies on correlation measures to relate gene expression profiles, genetic markers and clinical traits. Using a correlation measure affords a truly unified approach for relating variables from disparate data sets. We demonstrate IWGCNA on a chronic fatigue syndrome (CFS) data set and show that it identifies candidate genes whose functions are consistent with results from other CFS studies.

### Background on chronic fatigue syndrome

Chronic fatigue syndrome (CFS) is a major public health problem that affects more than one million people in the US [23]. CFS is defined as debilitating fatigue of at least six months duration accompanied by at least four of the following case defining symptoms: post exertional fatigue lasting longer than 24 hours, unrefreshing sleep, diffculty concentrating or remembering, headaches unusual in frequency or duration, muscle pain, joint pain, sore throat and tender lymph nodes [24]. CFS has been associated with similar debilitating conditions such as fibromyalgia, connective tissue disease and mitochondrial deficiency [25,26]. CFS has been shown to affect the endocrine, muscular and immune systems [27-29] and some cases may be triggered by viruses [30]. While there is no consistent cause, evidence for immune and hypothalamic-pituitary-adrenal (HPA) axis abnormalities have been observed at the symptom, molecular and genetic level of CFS patients [31].

Several groups have found higher cytotoxic T-cell counts and impaired T-cell function in CFS patients in comparison to controls [32,33]. There has also been compelling evidence for higher rates of immune cell apoptosis in CFS patients, specifically neutrophils and peripheral blood lymphocytes [34,35]. The HPA axis is a feedback system that mediates glucocorticoid hormones (cortisol) and serotonin and is closely linked to the immune system. It is thought that a dysfunctional HPA axis might be linked to CFS [31,36]. Subclasses of CFS have been associated with polymorphisms in genes that function in the HPA axis *NR3C1*, *TPH2 *and *MAOA *[37-39].

While molecular profiles and genetic variants within genes related to the immune system and the HPA axis have been shown to be associated with CFS [40-42] there is a need to gain a systems level understanding of the disease. Standard gene mapping techniques are not designed to identify pathways underlying complex traits, which exhibit genetic heterogeneity involving many small-effect genes. The quest to determine the genetic etiology of CFS is further obfuscated by diagnostic errors, phenotypic heterogeneity and in some cases environmental effects.

Recent systems genetic strategies that characterize interactions between genotype data and co-expression modules have successfully been applied to complex diseases [11,43]. Here we present the IWGCNA approach for integrating a weighted gene co-expression network with SNP data to identify a disease-related module and to develop a systems genetic gene-screening strategy that generates testable hypotheses. Furthermore, we use the Network Edge Orienting (NEO) software to show that this screening strategy selects genes that are causal for the module [18]. Our analysis identifies novel genes associated with CFS severity that are causal drivers for a severity-related module. Gene ontology software indicates that IWGCNA identifies clinically relevant biological pathways and genes.

## Results

**Figure 1:**
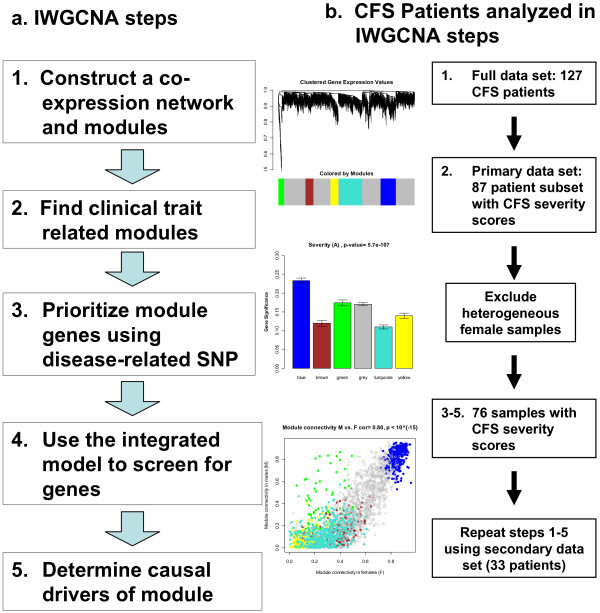
**a. Flow chart overview of methods and b. subsets of patients analyzed at each step**. We first constructed a co-expression network based on 127 CFS samples and then identified a CFS severity-related module using a subset of 87 patients with CFS severity scores. We then related the SNPs and connectivities to the module gene expressions in both the males and homogenized female samples separately. We selected candidate genes based on 1) association with a SNP that in turn was associated with severity, 2) connectivity, and 3) association with severity in both sexes. We then repeated analysis steps 1–5 on a second data set.

The fundamental tenets of IWGCNA are to find gene expressions that are 1) significantly related to the clinical trait, 2) highly connected "hub" genes in a disease related co-expression module and 3) significantly associated with a disease-related genetic marker. We apply this approach to a chronic fatigue syndrome (CFS) data set consisting of microarray, SNP, and trait data (CFS severity). As our analysis of this data set consists of several steps working with different subsets of data, we provide a flow chart overview in Figure 1 and begin with an outline of IWGCNA. We then present results from our analysis of the CFS data and compare them to the results obtained from a standard analysis approach that ignores the SNP data. Finally, we show that IWGCNA identifies functionally relevant candidate genes that are causal drivers for their trait-related parent module.

### Step 1: Construct a co-expression network and modules

We define co-expression networks as undirected, weighted gene networks. The nodes of such a network correspond to gene expression profiles, and edges between genes are determined by the pairwise correlations between gene expressions. Network construction was performed using our freely available customized R software functions [8-10,44]. The absolute value of the Pearson correlation coefficient is calculated for all pair-wise comparisons of gene-expression values across all microarray samples. The correlation matrix is then transformed into a weighted undirected network (i.e., a matrix of connection strengths) by raising the absolute value of each entry to a power *β*. High values of *β *emphasize high correlations at the expense of low correlations. Unlike unweighted networks that use a hard threshold to dichotomize the correlation matrix, the soft thresholding of weighted gene co-expression networks preserves the continuous nature of the gene co-expression information, leading to highly robust results and allowing for a simple geometric interpretation of network concepts [8,45,46].

The next step is to organize the genes into clusters or modules. Toward this end we use topological overlap, which is a robust measure of interconnectedness [47-49]. The (*i*, *j*) entry in the topological overlap matrix reflects a shared connectivity pattern between genes *x*_*i *_and *x*_*j*_. Average linkage hierarchical clustering is then used to cluster the genes into modules using the topological overlap dissimilarity measure [8,48]. Several centrality measures have been proposed in the literature [45,50]. Here we focus on centrality (connectivity) measures that are useful within the WGCNA context. Whole network connectivity *k*(*i*) is the sum of the connection strengths between a particular gene *x*_*i *_and all other genes in the network $k(i)=\sum_{j\in N,j\neq i}{\left|\text{Cor}(x_{i},x_{j})\right|}^{\beta }$, where *N *refers to the set of network genes. Intramodular connectivity *k*^*q*^(*i*) is another measure which is more meaningful for our module-based analysis. It is computed from the sum of the connection strengths between a particular gene and all other genes in the module $k^{q}(i)=\sum_{j\in q,j\neq i}{\left|\text{Cor}(x_{i},x_{j})\right|}^{\beta }$, where *q *refers to a specific module. Another measure of connectivity is the module eigengene-based connectivity $k_{ME}^{q}(i)$, which is computed from the absolute value of a gene expression *x*_*i *_within the *q*-th module and its first principal component or "*q*-th module eigengene", *ME*^*q*^. Specifically, $k_{ME}^{q}(i)$ = |Cor(*x*_*i*_, *ME*^*q*^)|, where larger values indicate greater similarity between a gene *x*_*i*_and the *q*-th module eigengene. One can show that the module eigengene-based connectivity measure is highly correlated with intramodular connectivity [45], but a theoretical advantage of $k_{ME}^{q}(i)$ is that its definition can be easily extended to expression profiles outside the module. Another advantage of $k_{ME}^{q}(i)$ is that a simple correlation test p-value can be used to assess the statistical significance of the relationship between *x*_*i *_and *ME*.

### Step 2: Find clinical trait-related modules

To incorporate external information into the co-expression network, we first define a measure of *gene significance *(*GS*). Abstractly speaking, the higher the *i*-th gene's |*GS*(*i*)|, the greater its biological significance. For example, *GS*(*i*) could encode pathway membership (e.g., 1 if the gene is a known apoptosis gene and 0 otherwise), knockout essentiality, or the correlation with an external microarray sample trait. A gene significance measure could also be defined by minus log of a p-value. The only requirement is that a gene significance of 0 indicates that the gene is not significant with regard to the biological question of interest.

We define *GS*_*severity*_(*i*) as the absolute value of the correlation between the CFS severity phenotype and the *i*-th gene expression *x*_*i*_: *GS*_*severity*_(*i*) = |Cor(*x*_*i*_, severity)|. A correlation test can be used to assign a statistical significance level (p-value) to *GS*_*severity*_(*i*). Note that a *β *power of gene significance, |Cor(*x*_*i*_, severity)|^*β*^, can be interpreted as the connection strength between severity and the *i*-th gene expression in a weighted network. To arrive at a measure of *module significance*, we average the *GS*_*severity *_values of all genes within a module. Alternatively, one could define a module significance measure by correlating the trait with the module eigengene [45]. Subsequent analyses focus on the module that is most related to the clinical trait of interest.

### Step 3: Prioritizing gene expressions with a SNP marker

This step requires a SNP marker that is associated with both the trait and the trait-related module. To measure the association between a SNP and the gene expression profiles we define a SNP-based gene significance measure *GS*_*SNP*_(*i*) = |Cor(*x*_*i*_, *SNP*)|. In our application we use a correlation test to compute the corresponding p-value for *GS*_*SNP*_(*i*). *GS*_*SNP *_is similar to a single point LOD score, as it measures the extent to which a gene is associated with the SNP.

### Step 4: Using network connectivity and genetic information to find candidate genes

While a standard gene screening approach would draft a final list of candidate genes based solely on the association between gene expression and the clinical trait (*GS*_*severity*_), our integrated screening strategy additionally uses *GS*_*SNP*_, and *k*_*ME*_. This approach allows us to select disease related genes that are implicated by the genetic marker and network connectivity information.

### Step 5: Network edge orienting analysis to determine causal drivers of module

We use the Network Edge Orienting (NEO) software to produce edge orienting scores which allow us to determine whether a candidate gene is causal or reactive to its parent module [18]. Since we use a single genetic marker as a causal anchor, we use the *LEO.NB.SingleMarker *score to evaluate the causal edge *x*_*i *_→ *ME*, where *x*_*i *_is the expression profile of the *i*-th candidate gene and *ME *is the module eigengene. Genes with a causal relationship to their parent module are highly related to many other genes within the module and are upstream of the module expressions.

The systems genetic analysis described in steps 1–5 results in a biologically motivated gene screening strategy. Pathway analysis and additional data sets can then be used to support and/or prioritize the resulting candidate genes.

### An IWGCNA of chronic fatigue syndrome

In the following sections, we apply the IWGCNA to a chronic fatigue syndrome (CFS) data set consisting of phenotype, genotype and expression data from the Centers for Disease Control [38,40,51]. The CFS patients studied here were a subset of a 227 patient cohort from Wichita, KS collected between December 2002 and July 2003 [52]. Details on the CFS severity measure as well as other diagnostic criteria are included in the Methods section.

#### Defining co-expression network modules and relating them to the CFS trait data

**Figure 2:**
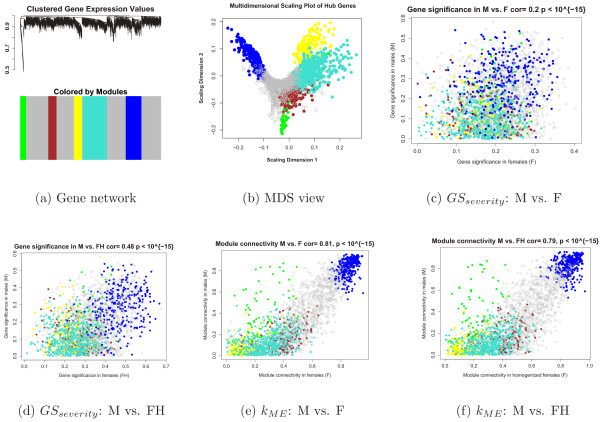
**Graphical representations of network properties**. (a) Hierarchical clustering of the 2677 most varying and connected genes resulted in five modules. (b) A multi-dimensional scaling plot of these genes indicates that the blue module is the most distinct. (c) There is little relationship between male and female gene expression correlations with CFS severity, likely due to genetic heterogeneity in the female samples. (d) Homogenizing the female samples more than doubled the correlation between M and FH gene significance. (e) Connectivities of the module genes are similar between males (M) and females (F) and (f) males and homogenized females (FH), with the blue module showing the highest preservation. The fact that intramodular connectivity is highly preserved forms the foundation of a connectivity and network-based screening strategy.

**Figure 3:**
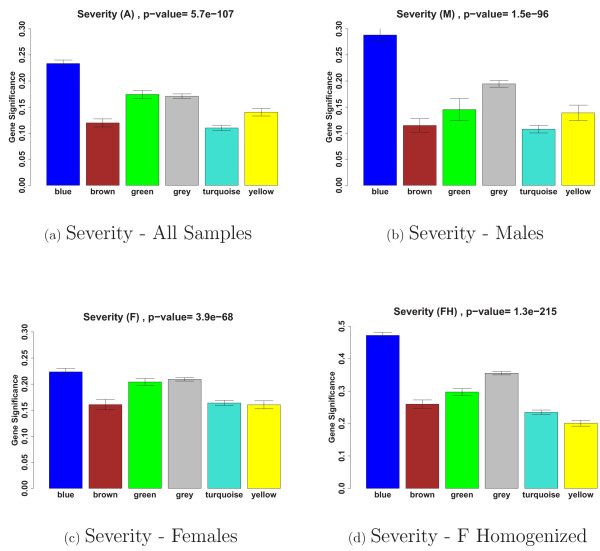
**Male and female gene significance bar plots for CFS severity**. We found that the blue module gene significance was highest in (a) all samples and in (b) males. In females (c) the blue module significance was approximately equal to the average significance of the other modules. (d) Homogenizing the female samples increased and emphasized the blue module significance.

Starting with the 8966 most varying genes (where "genes" refers to "probes") described in the Methods section, we selected the 30% most connected (2677) for our network analysis. WGCNA identified five modules of co-expressed genes. Figure 2(a) shows a cluster tree of the gene network, where the five color-coded modules correspond to branches of this tree. The color band underneath the tree depicts the branches (modules), and grey denotes the genes outside of the modules (background genes). A classical multi-dimensional scaling plot illustrates the relative positions of the module genes (Figure 2b). Next, we related our five modules to the severity trait to identify the module with the strongest association. Figure 3 shows that the blue module with 299 genes has the highest module significance in (a) all samples (mean *GS*_*severity *_= 0.234, corresponding to a p-value of 0.007), (b) males and (c) females. As a result, we focused on this module in the following analyses.

#### Modules facilitate a molecular characterization of gender differences

Since CFS is four times more likely to occur in women than in men [51], it is possible that there are genetic differences between men and women regarding CFS severity. Furthermore, women outnumber men three to one in this data set, so without considering gender in our analysis, results could be skewed toward severity related alleles that are more important in women. To ensure that our analysis produced gene candidates related to CFS severity in both sexes, we stratified the analysis by sex.

Before relating the blue module genes to severity and the SNP data, we investigated whether the network model was preserved when the data was stratified by sex. We compared the *GS*_*severity *_values between males and females (Figure 2c) and found a weak correlation (r = 0.197). However, the blue module was associated with CFS severity in both men and women independently and in the samples combined. Furthermore, there was a high correlation (r = 0.81; p-value < 10^-16^) between the $k_{ME}^{blue}$(*i*) values of the male and female networks (Figure 2e). The importance of the blue module in both genders and the preservation of the module membership measure $k_{ME}^{blue}$(*i*) demonstrate the value of using network properties to screen for genes.

Figure 3(b) shows that the blue module was the only module that was highly related to severity in males, whereas in females the blue module was only slightly more significant than the other modules 3(c). Because the relationship between gene expression and severity was stronger in males, we used the blue module eigengene to find a more genetically homogeneous female sample.

#### Using the module eigengene to homogenize the female samples

To eliminate heterogeneous samples from the female data set, we made use of the fact that the blue module eigengene (MEblue) was significantly correlated with CFS severity (r = 0.272; p-value = 0.011). Thus, patients whose severity score is inconsistent with the blue module eigengene expression are unlikely to be related to the associated disease pathway. We "homogenized" the female data set by restricting the analysis to samples with either a) moderate to high severity (severity > 1) that also had a high blue module eigengene value MEblue > mean(MEblue) or b) less severe samples (severity = 1) with MEblue < mean(MEblue). Homogenization excluded 11 samples, resulting in 53 homogenized female (HF) samples. As expected, homogenization increased the mean module significance from 0.223 (p-value = 0.074) to 0.472 (p-value = 1.6 × 10^-4^). Since homogenization amounts to sample selection, the resulting p-values are biased and should be interpreted as descriptive rather than inferential measures. Homogenization can be used to reduce the genetic heterogeneity inherent in complex trait studies.

#### Identifying a relevant SNP marker

The genetic marker data consisted of 36 autosomal SNPs located near or within a set of eight genes that were considered biologically relevant for CFS (see Methods section for details) [38]. We chose to focus on SNP rs10784941 located within the *TPH2 *tryptophan hydroxylase 2 gene because it had previously been shown to be associated with chronic fatigue, and it was associated with CFS severity in our data set [38,39]. Table 1 reports the average severity correlations for each of the eight genes and its most correlated SNP. The *TPH2 *SNP was associated with severity (p-value = 0.010) and moderately associated with the blue module gene expressions (p-value = 0.077). The *TPH2 *gene functions in serotonin synthesis which is part of the hypothalamic-pituitary-adrenal (HPA) feedback system that has been consistently implicated in CFS [53,54].

**Table 1:** Average absolute value of severity associations for the SNPs within eight candidate genes.

**Gene Name**	**Gene Location**	**Average Correlation (SD)**	**Count of SNPs in candidate gene**	**Most Correlated SNP**		
				Name	Correlation	p-value
*POMC*	2p24	0.14 (NA)	1	rs12473543	0.135	0.216
*NR3C1*	5q34	0.07 (0.06)	7	rs258750	0.198	0.069
*CRHR2*	7p15	0.15 (0.08)	3	hCV15960586	0.225	0.036
*TH*	11p15	0.07 (0.01)	2	rs4074905	0.080	0.466
*TPH2*	12q21	0.23 (0.04)	7	rs10784941	0.275	0.010
*SLC6A4*	17q11.1	0.18 (0.17)	3	rs4325622	0.347	0.001
*CRHR1*	17q21	0.03 (0.02)	6	rs242940	0.069	0.531
*COMT*	22q11.1	0.04 (0.02)	7	hCV11804654	0.077	0.479

*TPH2 *with seven SNPs had the highest average association with CFS severity.

Table 2 shows genetic correlations with the severity trait in five different subgroups of data and in a second data set (where the second data set is detailed in the Methods section). Severity was significantly correlated (p-value = 0.011) with the blue module eigengene in all samples and had moderate correlation in females. The severity association with the *TPH2 *SNP was very significant for all samples combined (p-value = 0.010), the male samples (p-value = 0.030), and moderately significant in the female samples after homogenization (p-value = 0.076). Since homogenization strengthened the relationship between severity and the *TPH2 *SNP in both the female samples and in the male and homogenized female samples combined, we used the homogenized samples in our gene screening procedure.

**Table 2:** Understanding the factors that affect gene significance.

**Data Set**	**No. Samples**	**a.) cor(severity, MEblue)^1^**	**b.) cor(severity, SNP)^2^**
Samples with severity scores	87	r = 0.27 (p = 0.011)	r = 0.28 (p = 0.010)
Males & HomFemales	76	r = 0.50 (p = 8 × 10-6)	r = 0.33 (p = 0.003)
Males with severity scores	23	r = 0.34 (p = 0.113)	r = 0.45 (p = 0.030)
Females with severity scores	64	r = 0.26 (p = 0.041)	r = 0.17 (p = 0.170)
Homogenized Females	53	r = 0.54 (p = 2 × 10-5)	r = 0.25 (p = 0.076)
Second data set (DS)^3^	33	r = 0.09 (p = 0.638)	r = 0.03 (p = 0.846)
Second DS Homogenized^3^	30	r = 0.38 (p = 0.040)	r = 0.15 (p = 0.415)

**Data Set**	**No. Samples**	**c.) cor(severity, *FOXN1*)**	**d.) cor(SNP, *FOXN1*)**

Samples with severity scores	87	r = 0.21 (p = 0.055)	r = 0.18 (p = 0.088)
Males & HomFemales	76	r = 0.38 (p = 6 × 10-4)	r = 0.23 (p = 0.045)
Males with severity scores	23	r = 0.27 (p = 0.216)	r = 0.20 (p = 0.365)
Females with severity scores	64	r = 0.21 (p = 0.101)	r = 0.19 (p = 0.137)
Homogenized Females	53	r = 0.44 (p = 0.001)	r = 0.24 (p = 0.082)
Second DS^3^	33	r = 0.28 (p = 0.116)	r = 0.18 (p = 0.318)
Second DS Homogenized^3^	30	r = 0.40 (p = 0.030)	r = 0.12 (p = 0.515)

The severity, MEblue, *TPH2 *SNP, and *FOXN1 *correlations (r) and p-values (p) for five different subsets of the first primary data set and the homogenized samples in the secondary data set. Severity is most significantly related toMEblue, the *TPH2 *SNP and the candidate *FOXN1 *gene in the combined male and homogenized female sub set. Homogenizing the second data set (which discarded three samples) also improved the MEblue and *FOXN1 *associations with severity.

^1^MEblue refers to the blue module eigengene, or first principal component of the blue module.

^2^SNP refers to the *TPH2 *SNP rs10784941.

^3^Here empiric replaces severity in cor(severity, *FOXN1*).

#### Systems genetic screening criteria

Because the connectivity $k_{ME}^{blue}$ can be interpreted as a measure of membership to the blue module, it can be used to prioritize pathway defining genes. We selected candidate genes that met the following criteria in both males and homogenized female samples: i) *GS*_*TPH*2 _greater than 0.2 to select genes that were associated with a CFS-related SNP, ii) $k_{ME}^{blue}$ in the top 80% to select genes that were centrally located within the blue module, and iii) *GS*_*severity *_and *GS*_*TPH*2 _signs that were consistent in both sexes. The purpose of this last criterion was to safeguard against potentially spurious correlations. We reasoned that genes which are positively correlated with severity in one sex but are negatively correlated in the other are less credible than those with strong correlations in the same direction among both sexes. iv) We also required a moderate correlation of 0.2 with the severity trait (*GS*_*severity*_) in males and a slightly stronger correlation of 0.35 in the homogenized females (since homogenization increased the *GS*_*severity *_measure). Out of the 2677 network genes, twenty met these four criteria: *C3ORF26*, *CD302*, *CRNKL1*, *DCTN2*, *FOXN1*, *LTV1*, *MED8*, *NPAL2*, *PBLD*, *PGK1*, *PPP1R14C*, *PRDX3*, *PRKCH*, *RYK*, *SNURF*, *SUCLA2*, *TFB2M*, *TMEM50A*, *VAMP5 *and *XM13557*. Annotation and correlation information are provided in Additional File 1 and Table 3, respectively. We found that selection of the *FOXN1 *gene was relatively robust with respect to the choice of screening criteria as long as association with the *TPH2 *SNP was imposed in both sexes.

**Table 3:** Pearson correlations (r) between the expression profiles of the 20 candidate genes from the IWGCNA and MEblue, CFS severity, and the *TPH2 *SNP.

	**Candidate Genes from IWGCNA: Gene Name and Genbank Accession**	**Pearson correlations with gene expression profiles**									
		MEblue		**CFS Severity**				*TPH2 *SNP			
		r: All	Rank*	**r: All**	**p-value**	**r: M**	**r: F**	r: All	p-value	r: M	r: F
1	FOXN1 (NM_003593)	0.845	195	**0.21**	**0.055**	**0.27**	**0.21**	0.21	0.018	0.23	0.20
2	PRDX3 (AF118073)	0.848	181	**0.26**	**0.017**	**0.43**	**0.21**	0.21	0.020	0.32	0.17
3	SUCLA2 (AK001458)	0.844	197	**0.20**	**0.059**	**0.25**	**0.18**	0.20	0.021	0.36	0.17
4	DCTN2 (NM_006400)	0.909	30	**0.18**	**0.087**	**0.21**	**0.16**	0.23	0.009	0.41	0.18
5	PGK1 (AB062432)	0.849	176	**0.22**	**0.045**	**0.37**	**0.21**	0.14	0.108	0.26	0.12
6	SNURF (AF101044)	0.882	77	**0.27**	**0.012**	**0.51**	**0.20**	0.18	0.037	0.32	0.14
7	PRKCH (BC001000)	0.888	64	**0.16**	**0.143**	**0.27**	**0.14**	0.15	0.089	0.23	0.13
8	RYK (NM_002958)	0.867	113	**0.21**	**0.048**	**0.26**	**0.20**	0.12	0.182	0.21	0.09
9	PPP1R14C (AF407165)	0.866	114	**0.24**	**0.025**	**0.21**	**0.25**	0.21	0.016	0.26	0.19
10	VAMP5 (AF077197)	0.863	124	**0.33**	**0.002**	**0.47**	**0.29**	0.24	0.007	0.35	0.20
11	PRO0641 (AF090939)	0.853	159	**0.32**	**0.003**	**0.43**	**0.30**	0.21	0.016	0.25	0.19
12	TMEM50A (AF081282)	0.911	23	**0.26**	**0.014**	**0.29**	**0.26**	0.17	0.050	0.22	0.16
13	CRNKL1 (AF111802)	0.865	117	**0.27**	**0.012**	**0.41**	**0.23**	0.22	0.013	0.36	0.18
14	NPAL2 (AK024017)	0.919	10	**0.35**	**0.001**	**0.37**	**0.35**	0.21	0.020	0.33	0.16
15	TFB2M (AK026314)	0.899	49	**0.31**	**0.004**	**0.29**	**0.31**	0.21	0.016	0.22	0.19
16	PBLD (AK027673)	0.906	38	**0.31**	**0.003**	**0.28**	**0.34**	0.17	0.049	0.23	0.15
17	LTV1 (AK027815)	0.856	145	**0.30**	**0.005**	**0.39**	**0.29**	0.19	0.029	0.29	0.17
18	MED8 (BC010019)	0.869	108	**0.29**	**0.007**	**0.31**	**0.28**	0.22	0.015	0.35	0.18
19	CD302 (BC020646)	0.817	315	**0.24**	**0.028**	**0.28**	**0.25**	0.18	0.046	0.29	0.16
20	(XM13557)	0.887	68	**0.32**	**0.002**	**0.36**	**0.29**	0.19	0.032	0.38	0.15
	**Median:**	**0.866**	**114**	**0.27**	**0.013**	**0.30**	**0.25**	**0.21**	**0.021**	**0.29**	**0.17**

The correlations were computed using all 127 samples studied (All), the 98 female samples and the 29 male samples except for the correlation with severity which only had 87 non-missing scores (64 female and 23 male). The CFS Severity column is bolded for clarity.

*The rank of each gene in terms of its MEblue correlation out of the 8966 genes used to start the analysis.

When we applied these screening criteria to the 8966 most varying genes, 89 met these criteria and the gene names and correlation measures are provided (see Additional File 2). Note that all of the 20 candidate genes are included in this list.

#### Investigating causal relationships

Our next step was to orient relationships between the candidate genes and the severity-related module. Toward this end, we used the trait-related *TPH2 *SNP as a causal anchor in the Network Edge Orienting (NEO) software [18]. We defined a gene as being causal for the module if the *LEO.NB.SingleMarker *score for the causal model was positive and at least twice as probable as the maximum alternative model's score, i.e. we required a minimum *LEO.NB.SingleMarker *score of 0.30 ≈ *log*_10_(2). While a threshold of *log*_10_(10) = 1 was recommended by [18], we relaxed it here due to our small sample size (127 patients).

The *LEO.NB.SingleMarker *scores for the 20 candidate genes are provided in Additional File 1. There were 66 causal genes out of 299 blue module genes. All but three of our 20 candidate genes were causal for the blue module, with an average causality score rank of 25. A NEO analysis of the male and homogenized female data subset (76 samples) indicated that all but two of the 20 candidate genes were causal, with an average causality rank of 39 (not shown). These results indicate that our 5-step strategy identifies a trait-related module and its potential causal drivers.

#### Applying our gene screening strategy to a second data set

**Figure 4:**
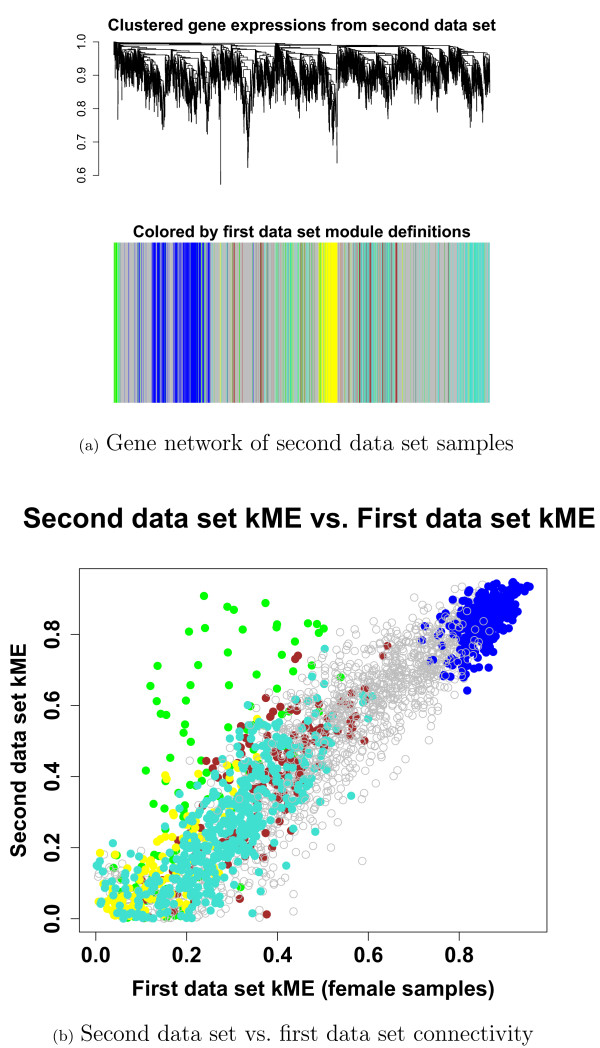
**Secondary data set results**. (a) Average linkage hierarchical clustering of the gene expressions from 33 secondary data set samples colored by the original network module definitions shows that the blue module is preserved. (b) Intramodular connectivity is preserved between the secondary and primary data set networks.

We applied our gene screening strategy to the 33 patient samples that were missing severity scores but had a similar measure of CFS severity called "empiric severity". The rationale was that replicating the candidate gene findings in these samples would support the IWGCNA results. We first checked that the module definitions from the first data set were preserved in the second data set. Figure 4(a) shows that the blue module was well preserved and Figure 4(b) shows that the corresponding module membership measures $k_{ME}^{blue}$ were preserved as well. Applying the same integrated gene screening criteria as described above resulted in 61 candidate genes, six of which had been identified in the primary data set: *FOXN1*, *DCTN2*, *PPP1R14C*, *VAMP5*, *TFB2M *and *XM13557*.

#### Pathway annotation of candidate genes

Additional File 1 includes pathway annotations for the 16 candidate genes that were eligible for annotation with Ingenuity^® ^Systems' Pathways Analysis (IPA, ) software. Column (a) gives results for an IPA analysis of the candidate genes, and (b) shows their corresponding annotations when the 299 blue module genes were analyzed (where 212 of the blue module genes were eligible for pathway annotation in August 2008). Out of the 16 candidate genes, IPA identified a highly significant pathway (p-value ≈ 10^-32^) containing 12 of them *FOXN1*, *PRDX3*, *SUCLA2*, *TFB2M*, *MED8*, *SNURF*, *DCTN2*, *PGK1*, *PRKCH*, *RYK*, *VAMP5 *and *PBLD*, and this pathway most likely functioned in Cell Cycle, Cancer, Cell Death, and Hematological Disease (p-value range = 1.15 × 10^-5^, 1.03 × 10^-1^). Column (b) shows that the 212 blue module analysis suggested functionally relevant pathways for the candidate genes such as i) Endocrine System Disorders, Infectious Disease, and Inflammatory Disease; ii) Connective Tissue Development and Function and iii) Viral Function. Pathways i-iii and hematological disease are consistent with results from previous CFS research [35,55-58].

We investigated the *TPH2 *SNP's contribution to our gene screening strategy by repeating the candidate gene IPA with *TPH2 *included. Indeed, IPA positioned *TPH2 *within the top hematological disease pathway containing 12 candidate genes. This finding supports the notion that SNP-associated gene expression profiles are likely to interact with the SNP-containing gene.

**Figure 5:**
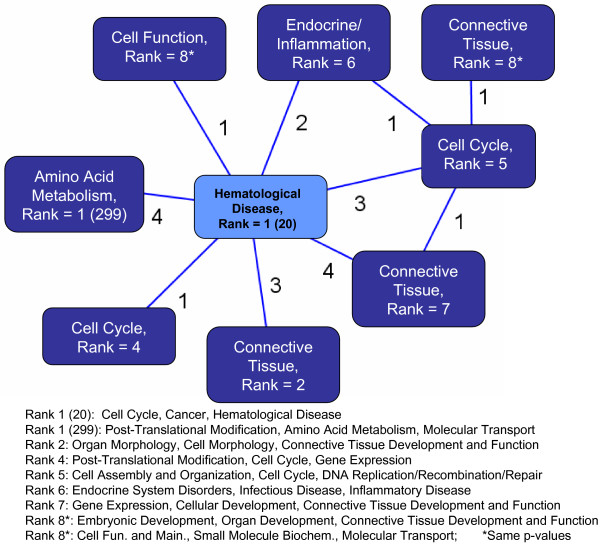
**Ingenuity Pathway Analysis results**. An IPA comparison analysis indicates that the 20 candidate gene pathway (light blue) is connected with several of the most highly significant blue module pathways (dark blue). Each pathway description was selected from the top three most significant IPA pathway annotations, and the other two are listed below the diagram. The ranks correspond to the p-values of the identified networks, where the network with the smallest p-value has rank = 1.

To determine known interactions between the candidate genes within the blue module, we carried out an IPA comparison between the candidate gene and blue module gene networks. Figure 5 shows that the main hematological disease pathway in the candidate gene IPA is directly connected to seven pathways within the blue module network (where the number of common genes are listed adjacent to the connection edges). This illustrates the value of IWGCNA: it identifies a candidate gene pathway that is centrally located within the blue module network, i.e. it identifies genes influencing multiple biological pathways.

#### Functional annotation of candidate genes

The IWGCNA of a CFS data set identifies candidate genes that interact in biologically relevant immune and connective tissue pathways. In this section, we show that in addition to belonging to relevant pathways, our candidate genes have functions that are consistent with findings from other CFS studies. Here we focus on *FOXN1*, *PRDX3*, and *SUCLA2*, but other interesting candidates are described in Additional File 1. *FOXN1 *is highly expressed in thymus epithelia cells. The thymus gland plays a pivotal role in the immune system by converting lymphocytes to T-cells and releasing functional T-cells to combat infection. A *FOXN1 *knockout mouse model has been shown to have a deficient immune system due to a lack of functional T-cells [59-61]. Similarly, humans with mutations in *FOXN1 *have an immune system deficiency [62,63]. Under the assumption that a compromised immune system can cause chronic fatigue, this knockout mouse suggests a potential role for *FOXN1 *in chronic fatigue. Because of its statistical significance and biological importance, *FOXN1 *is a candidate for investigating the immune system's role in CFS severity.

*PRDX3 *is a clinically interesting candidate because of its role in mitochondrial function and apoptosis. Specifically, it regulates the abundance of *H*_2_*O*_2 _and other reactive oxygen compounds that mediate apoptosis [64,65]. *SUCLA2 *is another gene involved in mitochondrial function that could be clinically relevant for chronic fatigue. Mutations in *SUCLA2 *have previously been associated with mitochondrial encephalomyopathy, a disorder which causes fatigue and muscle weakness [66,67]. These immune, cell death, and muscular system functions are consistent with findings from other CFS studies [27,28,34].

### A standard analysis of chronic fatigue syndrome that excludes the TPH2 SNP marker and module membership

IWGCNA requires at least one reliable SNP marker that is associated with the disease. While the relationship between CFS and the *TPH2 *SNP has been reported in a previous study, the relatively unimpressive p-value suggests that additional data are needed to confirm its validity. Here we present results from a standard analysis of the chronic fatigue data that excludes the SNP and module membership information.

**Figure 6:**
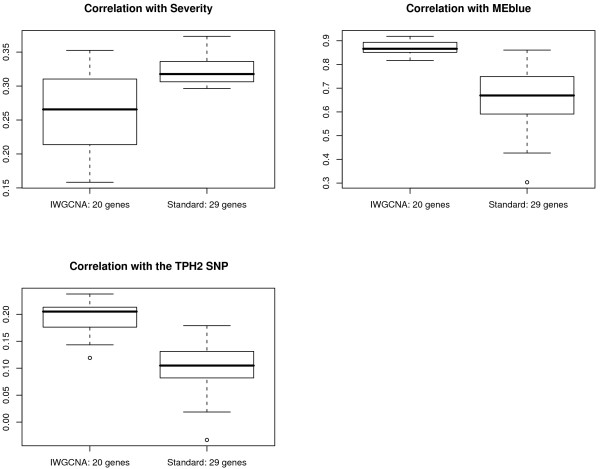
**Boxplot comparisons of correlation distributions for the 20 candidate genes from the IWGCNA and the 29 candidate genes from the standard analysis**. The correlations with severity are higher among the standard analysis candidate genes, but the MEblue and TPH2 SNP correlations are higher for the IWGCNA candidates.

Starting with the 8966 most varying genes, we computed the p-values for the Pearson correlation test of the gene expression profiles with the severity trait. For each p-value, we computed the corresponding local false discovery rate (q-value) using the qvalue package in R [68]. We used Ingenuity Pathways Analysis software to study pathways and functions of the 346 genes that achieved the minimum false discovery rate of 0.081. Among the 241 genes that were eligible for Ingenuity network construction, the top pathways were: 1) Viral Function, Molecular Transport, RNA Trafficking (p-value ≈ 10^-52^, focus molecules = 29); 2) Connective Tissue Development and Function, Cell Signaling, Molecular Transport (p-value ≈ 10^-31^, focus molecules = 20); and 3) Cell Morphology, Cellular Assembly and Organization, Cancer (p-value ≈ 10^-29^, focus molecules = 19). As the Viral Function pathway achieves the highest score and is clinically relevant to CFS, we consider these 29 genes as top candidates of the standard analysis. Table 4 gives the gene names and functional summaries for these genes. The *LEO.NB.SingleMarker *scores were excluded as only AF121255 with a score of 0.319 exceeded our causality threshold. We also present the correlations between these 29 genes and CFS severity, MEblue, and the *TPH2 *SNP in Table 5. Figure 6 indicates that the standard analysis genes tend to have higher correlations with severity than the IWGCNA genes. Also as expected, these genes tend to have lower correlations with MEblue and the *TPH2 *SNP than the IWGCNA genes.

**Table 4:** Candidate gene names and Entrez Gene descriptions from the standard analysis.

**Gene Name and Genbank Accession**		**Gene name. Entrez Gene and/or GeneRIFs description. Chromosome Location**
1	DGCR8 (AF165527)	DiGeorge syndrome critical region gene 8. 22q11.2

2	PPARD (BC002715)	Peroxisome proliferator-activated receptor delta. May be involved in the development of several chronic diseases, including diabetes, obesity, atherosclerosis, and cancer. 6p21.2-p21.1

3	IHPK2 (BC004469)	Inositol hexaphosphate kinase 2. May affect the growth suppressive and apoptotic activities of interferon-beta in some ovarian cancers. 3p21.31

4	CCDC92 (AB015292)	Coiled-coil domain containing 92. 12q24.31

5	NR5A2 (AB019246)	Nuclear receptor subfamily 5, group A, member 2. May be involved in regulation of Hepatitis B virus. 1q32.1

**6**	**PDPK1 **(BC006339)	**3-phosphoinositide dependent protein kinase-1. 16p13.3**

7	NXF1 (AF112880)	Nuclear RNA export factor 1. Exports viral mRNA's and herpes simplex virus type 1. 11q12-q13

8	COL13A1 (NM_080804)	Collagen, type XIII, alpha 1. May function in connective tissues. 10q22

9	AXIN2 (AF078165)	Regulates stability of beta-catenin in the Wnt signaling pathway. Mutations associated with colorectal cancer. 17q23-q24

10	SCAP (AK075528)	SREBF chaperone. Involved in regulating sterol biosynthesis. 3p21.31

11	DFFA (AF087573)	DNA fragmentation factor, 45 kDa, alpha polypeptide. Triggers DNA fragmentation during apoptosis. 1p36.3-p36.2

12	TCF4 (M74719)*	Transcription factor 7-like 2 (T-cell specific, HMG-box). Implicated in blood glucose homeostasis. 10q25.3

13	WNT16 (NM_016087)	Wingless-type MMTV integration site family, member 16. Implicated in oncogenesis and in several developmental processes. 7q31

14	ZNF687 (BC032463)	Zinc finger protein 687. 1q21.2

15	FGF1 (BC032697)	Fibroblast growth factor 1 (acidic). Embryonic development, cell growth, morphogenesis, tissue repair, tumor growth and invasion. 5q31

16	ANKRD6 (BC001078)*	Ankyrin repeat domain 6. 6q14.2-q16.1

17	EPHX1 (M36374)	Epoxide hydrolase 1, microsomal (xenobiotic). Activation and detoxification of exogenous chemicals such as polycyclic aromatic hydrocarbons. 1q42.1

18	FAIM (AK001444)	Fas apoptotic inhibitory molecule. 3q22.3

**19**	**ZMYND11 **(NM_006624)	**Zinc finger, domain containing 11. Binds adenovirus E1A protein. 10p14**

20	ADFP (NM_001122)	Adipose differentiation-related protein. 9p22.1

21	BAT5 (BC031839)	HLA-B associated transcript 5. Possibly involved in immunity. 6p21.3

22	CEBPA (NM_004364)	CCAAT/enhancer binding protein, alpha. Body weight homeostasis. 19q13.1

23	HNRNPA1 (NM_002136)	Heterogeneous nuclear ribonucleoprotein A1. May be part of the regulatory mechanisms of the life cycle of HTLV-1 human retrovirus in T cells. 12q13.1

**24**	**DMBT1 **(NM_004406)	**Deleted in malignant brain tumors 1. May play a role in the interaction of tumor cells and the immune system. 10q25.3-q26.1**

25	RNASEN (AF116910)	Ribonuclease type III, nuclear. Participates in diverse RNA maturation and decay pathways. 5p13.3

26	EDAR (AF130988)	Ectodysplasin A receptor. Mutations in this gene result in hypohidrotic ectodermal dysplasia. 2q11-q13

27	F3 (AF540377)	Coagulation factor III (thromboplastin, tissue factor). Enables cells to initiate the blood coagulation cascades. 1p22-p21

28	HSPG2 (AL445795)	Heparan sulfate proteoglycan 2. 1p36.1-p34

**29**	**EIF2C2 **(AF121255)	**Eukaryotic translation initiation factor 2C, 2. Encodes a member of the Argonaute family of proteins which play a role in RNA interference. 8q24**

*Members of the blue module.

**Table 5:** Pearson correlations (r) between the expression profiles of the 29 candidate genes from the standard analysis and MEblue, severity, and the *TPH2 *SNP.

	**Candidate Genes from Standard Gene Name and Genbank Accession**	**Pearson correlations with gene expression profiles**									
		MEblue		**CFS Severity**				*TPH2 *SNP			
		r: All	Rank^1^	**r: All**	**p-value**	**r: M**	**r: F**	r: All	p-value	r: M	r: F
1	DGCR8 (AF165527)	0.81	329	**0.37**	**<0.001**	**0.35**	**0.39**	0.13	0.14	0.12	0.12

2	PPARD (BC002715)	0.54	2305	**0.37**	**0.001**	**0.49**	**0.34**	0.10	0.25	0.18	0.08

3	IHPK2 (BC004469)	0.48	2842	**0.36**	**0.001**	**0.36**	**0.36**	0.15	0.09	0.02	0.16

4	CCDC92 (AB015292)	0.43	3434	**0.35**	**0.001**	**0.33**	**0.38**	0.07	0.42	-0.07	0.10

5	NR5A2 (AB019246)	0.47	2945	**0.35**	**0.001**	**0.37**	**0.32**	0.10	0.27	0.18	0.07

**6**	**PDPK1 **(BC006339)	**0.67**	**1218**	**0.35**	**0.001**	**0.47**	**0.31**	**0.16**	**0.08**	**0.24**	**0.12**

7	NXF1 (AF112880)	0.63	1517	**0.34**	**0.001**	**0.17**	**0.36**	0.15	0.10	-0.03	0.15

8	COL13A1 (NM_080804)	0.51	2590	**0.34**	**0.001**	**0.25**	**0.34**	0.05	0.60	-0.10	0.06

9	AXIN2 (AF078165)	0.80	414	**0.33**	**0.002**	**0.39**	**0.32**	0.13	0.15	0.16	0.10

10	SCAP (AK075528)	0.64	1458	**0.33**	**0.002**	**0.44**	**0.36**	0.17	0.06	0.38	0.12

11	DFFA (AF087573)	0.71	948	**0.33**	**0.002**	**0.25**	**0.35**	0.11	0.21	0.07	0.10

12	TCF4 (M74719)	0.76	637	**0.33**	**0.002**	**0.38**	**0.31**	0.14	0.13	0.15	0.11

13	WNT16 (NM_016087)	0.71	910	**0.33**	**0.002**	**0.38**	**0.32**	0.08	0.36	0.13	0.05

14	ZNF687 (BC032463)	0.83	243	**0.33**	**0.002**	**0.53**	**0.30**	0.10	0.28	0.29	0.04

15	FGF1 (BC032697)	0.65	1378	**0.32**	**0.003**	**0.26**	**0.31**	0.02	0.83	-0.03	0.00

16	ANKRD6 (BC001078)	0.83	241	**0.31**	**0.003**	**0.36**	**0.28**	0.14	0.12	0.23	0.09

17	EPHX1 (M36374)	0.64	1442	**0.31**	**0.003**	**0.24**	**0.33**	0.11	0.22	-0.01	0.12

18	FAIM (AK001444)	0.86	132	**0.31**	**0.004**	**0.34**	**0.30**	0.07	0.43	0.11	0.04

**19**	**ZMYND11 **(NM_006624)	**0.67**	**1223**	**0.31**	**0.004**	**0.47**	**0.26**	**0.13**	**0.15**	**0.32**	**0.07**

20	ADFP (NM_001122)	0.69	1057	**0.31**	**0.004**	**0.27**	**0.32**	0.11	0.20	0.12	0.10

21	BAT5 (BC031839)	0.73	802	**0.31**	**0.004**	**0.27**	**0.31**	0.10	0.24	0.08	0.09

22	CEBPA (NM_004364)	0.70	980	**0.31**	**0.004**	**0.16**	**0.32**	0.08	0.38	-0.16	0.10

23	HNRNPA1 (NM_002136)	0.46	3048	**0.30**	**0.004**	**0.23**	**0.34**	0.11	0.23	-0.12	0.15

**24**	**DMBT1 **(NM_004406)	**0.59**	**1874**	**0.30**	**0.005**	**0.50**	**0.24**	**0.08**	**0.37**	**0.20**	**0.03**

25	RNASEN (AF116910)	0.75	680	**0.30**	**0.005**	**0.29**	**0.31**	0.10	0.26	0.09	0.08

26	EDAR (AF130988)	0.82	288	**0.30**	**0.005**	**0.20**	**0.33**	0.09	0.32	-0.02	0.09

27	F3 (AF540377)	0.67	1181	**0.30**	**0.005**	**0.28**	**0.30**	0.10	0.26	0.18	0.06

28	HSPG2 (AL445795)	0.30	4880	**0.30**	**0.005**	**0.09**	**0.33**	-0.03	0.71	-0.25	-0.02

**29**	**EIF2C2 **(AF121255)	**0.60**	**1760**	**0.30**	**0.005**	**0.29**	**0.27**	**0.18**	**0.04**	**0.27**	**0.13**

	**Median^2^:**	**0.67**	**1218**	**0.32**	**0.003**	**0.33**	**0.32**	**0.10**	**0.24**	**0.13**	**0.09**

The correlations were computed using the 127 samples studied (All), the 98 female samples and the 29 male samples except for the correlation with severity which only had 87 non-missing scores (64 female and 23 male). Four of the candidate genes indicated in bold satisfied our IWGCNA screening criteria (excluding blue module membership). The CFS Severity column is bolded forclarity.

^1^The rank of each gene in terms of its MEblue correlation out of the 8966 genes used to start the analysis.

^2^The median was computed using the absolute value.

Recall that an Ingenuity Pathways Analysis (IPA) of the 20 IWGCNA candidate genes and the *TPH2 *gene produced a top IPA network that included the *TPH2 *gene. For comparison we repeated this analysis using the 29 standard analysis genes and *TPH2*. Neither of the two resulting IPA networks contained the *TPH2 *gene, which is consistent with the low correlations observed between the *TPH2 *SNP and these genes. While both the standard analysis and IWGCNA identified viral function and connective tissue genes, there was no overlap between the top 20 IWGCNA and the top 29 standard analysis candidate genes. This result is not surprising since different methods were used to reduce the 8966 gene set to about 0.3% of its original size (20–29 genes). To provide a more comprehensive comparison, we applied the IWGCNA screening criteria to the 8966 gene set which resulted in 89 genes, including the top 20 IWGCNA genes (see Additional File 2). Four of the standard analysis genes were on this 89-gene list: *PDPK1*, *ZMYND11*, *DMBT1 *and *EIF2C2 *(Tables 4 and 5). Furthermore, nine of the standard analysis genes could be considered as part of the blue module since their module membership values were higher than the minimum $k_{ME}^{blue}$ = 0.722 of the 299 module genes.

## Discussion

We present a systems genetic screening method for identifying candidate complex disease genes when gene expression, genetic marker and clinical outcome data are available. We demonstrate IWGCNA in a set of patients who had been diagnosed with some fatigue symptoms according to the 1994 CFS case definition criteria. The IWGCNA identifies a CFS severity-related module consisting of 299 genes and a subset of 20 candidate genes within this module that hold particular promise for future CFS studies. In addition to belonging to a severity-related module the 20 IWGCNA candidate genes also a) had a high association with the *TPH2 *locus, b) high intramodular connectivity, and c) were related to CFS severity in both sexes. Genetic marker based causality analysis indicated that 17 of the 20 candidate genes were causal for their parent module (out of 66 total causal genes for the blue module). Furthermore, we found that the blue module and intramodular gene connectivities were highly preserved in a second set of samples that had a similar empiric diagnosis of CFS severity. Applying IWGCNA in this data set replicated six of our 20 candidate genes. Pathway annotation with IPA software showed that our candidate gene results agreed with previously published findings that CFS affects the endocrine, immune and connective tissue systems [35,55-57,69].

A standard gene-screening strategy based on the local false discovery rate (q-value) and IPA software suggested 29 genes that functioned in a viral pathway. Relative to the standard analysis candidates, the 20 IWGCNA genes had a moderate association with severity, and stronger associations with the *TPH2 *SNP and MEblue. IPA results showed that using a SNP marker to screen for candidate gene expressions can identify genes that are known to interact with the SNP-containing gene.

Although the candidate gene findings from the IWGCNA and standard analysis are compelling, our purpose here is to illustrate a novel systems genetic gene-screening method. While it is reassuring that IPA software suggested viral and connective tissue function for both the IWGCNA and standard analysis candidates, there was no overlap between the corresponding top 20 and top 29 candidate gene lists. A more comprehensive comparison revealed that four genes (*PDPK1*, *ZMYND11*, *DMBT1 *and *EIF2C2*) were implicated by both analyses. Furthermore, although the reported p-values and causality scores are useful for exploring relative gene significance, the actual values did not reach genome-wide significance. Although this is to be expected for a complex disease study of modest magnitude (here the number of samples varied between 87 and 127), it emphasizes a conservative interpretation of these results. Finally, the patient sample may not be representative of the typical CFS patient population, as these patients were physically able to attend clinic (although to our knowledge this problem is inherent in many CFS studies). We should also point out that the candidate genes relate to CFS severity among patients with some fatigue symptoms, so these genes may not distinguish CFS patients from healthy controls. In order to make a clinical contribution to CFS etiology, our candidate gene findings require validation in additional studies. The purpose of this article is to illustrate a systems genetic gene screening strategy that yields testable hypotheses for future investigations. IWGCNA is a step towards the development and application of systems genetic approaches to complex disease gene mapping.

## Conclusion

Integrating gene co-expression networks with allelic association studies holds great promise for elucidating the genetic basis of complex diseases. We describe an intuitive and simple five-step incarnation of such an approach (IWGCNA) and apply it to a chronic fatigue syndrome data set. Our complete R statistical software code is available at .

## Methods

We analyzed the phenotype, genotype and expression data from a four year longitudinal study conducted by the Centers for Disease Control (CDC) [38,40,51,52]. Of the 164 patients described in Reeves et al. [52], we focused on the 127 that were diagnosed with some fatigue according to the Intake diagnosis defined below (i.e., we removed the controls).

### CFS phenotypes: severity and empiric

The phenotype data included several variables that measured different aspects of chronic fatigue syndrome. "Intake diagnosis" was a 5-level classification of CFS based on the 1994 case definition criteria [24]. In addition to intake diagnosis, the data set included scores from established diagnostic procedures used to evaluate quality of life in people suffering from cancer and other illnesses: 1) Medical Outcomes Survey Short Form (SF-36), 2) Multidimensional Fatigue Inventory (MFI), and 3) CDC Symptom Inventory Case Definition scales [52,70]. The SF-36 scale assesses eight characteristics: physical function, role physical, bodily pain, general health, vitality, social function, role emotional, and mental health. The MFI scale assesses five characteristics: general fatigue, physical fatigue, mental fatigue, reduced motivation, and reduced activity. The CDC symptom inventory scale assesses symptoms accompanying chronic fatigue. Each of these 14 characteristics is derived from several scores designed to evaluate the particular characteristic. Reeves et al. (2005) clustered these scores from 118 patients and identified three clusters of CFS severity: high, moderate and low.

The analyses in this manuscript mostly focus on the CFS severity trait in a subset of patients who had some fatigue symptoms according to the intake diagnosis. We also analyzed a second set of patients who did not have severity scores but did have a similar measure of severity based on some of the scores used to define CFS severity "empiric severity". The empiric severity diagnosis was highly correlated with CFS severity (r = 0.782, p-value = 2.2 × 10^-16^).

### Primary and secondary data set subjects

The full data set consisted of 127 samples classified as ill according to the intake diagnosis. The majority were female (98), and about 95% were Caucasian. None of these CFS patients had an additional medical or pyschological condition that can be considered exclusionary [52,71].

We divided the full data set into two subsets according to a) patients with CFS severity scores available (87 total: 64 females and 23 males) and b) patients without severity scores who had empiric severity scores (39 total: 33 females and 6 males). The data set with severity measures was the main data set analyzed in this manuscript and we refer to it as the primary or first data set. The primary data set had the following CFS severity distribution: high (24), moderate (48), and low (15). We refer to the remaining data samples as the secondary or second data set and we use it to support our primary data findings. Since the majority of the second data set samples were female, we avoided sex confounding by analyzing only the female subjects in this data set. The resulting secondary data set consisted of empiric severity, gene expression and SNP data for 33 female samples.

### Gene Expression Microarray Data

Peripheral blood mononuclear cells were assayed with approximately 20,000 probes from glass-slide arrays by MWG Biotech. ArrayVision software read the slides and normalized the data by subtracting background intensity from the spot intensity values. When background intensity exceeded spot intensity, ArrayVision set the probe intensity values to zero.

We excluded two outlier arrays based on their high mean gene expression levels and then using the remaining 162 arrays we filtered for genes whose mean expression was in the upper 50% and whose variance was in the upper 66%. These filtering criteria resulted in 8966 genes. Our remaining analyses focused on the 127 samples that had been classified as having fatigue symptoms according to the intake diagnosis. Additional gene filtering is described in the Results section.

### Genetic Marker Data

We considered 36 autosomal SNPs that the CDC had selected from eight candidate CFS genes, *TPH2 *(SNPs selected from locus 12q21), *POMC *(2p24), *NR3C1 *(5q34), *CRHR2 *(7p15), *TH *(11p15), *SLC6A4 *(17q11.1), *CRHR1 *(17q21), *COMT *(22q11.1) [38]. We additively coded the SNPs as 0, 1, or 2, for genotypes AA, AB, and BB, respectively. While this additive coding method may be sub-optimal for dominant or recessively acting loci, it has been shown to be effective for many genetic models.

### Causality analysis with the Network Edge Orienting software

We used a trait-related SNP marker as a causal anchor for the Network Edge Orienting (NEO) software to characterize whether each candidate gene expression was causal or reactive to the module eigengene (*ME*) [18]. We calculated the *LEO.NB.SingleMarker *(*LEO*) score, which is a relative fitting index that compares the model fitting p-value of the causal model for a gene *x*_*i *_causing *ME *to that of the next best competing model. For the edge orientation *x*_*i *_→ *ME*, the *LEO.NB.SingleMarker *score is given by

$$\begin{array}{r} LEO(x_{i}\to ME|SNP)=\\ \log_{10}\left(\frac{p(\text{model }1:SNP\to x_{i}\to ME)}{\max\left(\begin{array}{c} p(\text{model }2:SNP\to ME\to x_{i}),\\ p(\text{model }3:x_{i}\leftarrow SNP\to ME),\\ p(\text{model }4:SNP\to x_{i}\leftarrow ME),\\ p(\text{model }5:SNP\to ME\leftarrow x_{i}) \end{array}\right)}\right), \end{array}$$

where the competing models have the following interpretations model 2 implies that *ME *causes *x*_*i*_, model 3 implies that the *SNP *directly affects both *x*_*i *_and *ME *so that given the *SNP *they are independent of each other (confounded model), model 4 implies that the *SNP *and *ME *both affect *x*_*i *_and model 5 implies that the *SNP *and *x*_*i *_both affect *ME*. Although NEO performs well in simulation studies and several real data applications [18], we note that it has several limitations. The first limitation is that it requires the availability of genetic markers that are significantly associated with at least one trait per edge. Spurious associations between the markers and traits will result in meaningless edge orienting scores. The second limitation is that the structural equation model (SEM)-based edge orienting scores assume linear relationships between traits and SNP markers. This is mathematically convenient and allows the NEO approach to work in the domain of linear graphical models since it is based on correlations and SEMs. The third limitation is that causal inference and structural equation modeling assume that relevant traits and causal anchors have been included in the causal model. Under-specified causal models, i.e. models that omit important variables, may mislead the user to detect spurious causal relationships.

### Pathway Annotation Software

Ingenuity Pathways Analysis (IPA) software allowed us to compare co-expression interactions with interaction information that was manually curated from the literature and to annotate these interactions with the closest matching biological functions. The user-input or "focus" gene list was compared to the "Global Molecular Network" (GMN) database consisting of thousands of genes and interactions. The focus genes were sorted based on highest to lowest connectivities within the GMN, and then networks of approximately 35 genes were grown starting with the most connected focus gene. IPA creates networks based on the principle that highly connected gene networks are most biologically meaningful. It assigns a p-value for a network of size *n *and an input focus gene list of size *f *by calculating the probability of finding *f *or more focus genes in a randomly selected set of *n *genes from the GMN. Since these p-values are generally small, the -*log*_10_(p-value) "p-score" is reported. Similarly, a Fisher exact test p-value is calculated for the functional analysis. In this case the four categories include genes associated/not associated with the annotation and focus/non-focus genes. The IPA p-values were not corrected for multiple testing, and the authors recommend them as rough guides for approximating molecular function . The IPA interaction database is manually curated by scientists and updated quarterly. The results presented here were obtained in August 2008.

## Data and software availability

The complete chronic fatigue syndrome gene expression, genotype and clinical data, and R statistical software code for the IWGCNA presented here can be found at .

## Authors' contributions

APP and SH developed the methods and wrote the article. APP and CJS analyzed the data. MSR, SDV, EMS, and JCP revised the manuscript.

The Centers for Disease Control (CDC) collected the chronic fatigue gene expression, SNP marker and clinical trait data. At the CDC, TW supervises the genomics and proteomics laboratory in the Molecular Epidemiology Program in the Chronic Viral Diseases Branch; MSR supervises the genetics laboratory in the Molecular Epidemiology Program in the Chronic Viral Diseases Branch; SDV is Team Leader of the Molecular Epidemiology Program in the Chronic Viral Diseases Branch. All authors have approved the manuscript.

## Supplementary Material

Additional file 1 **Functional annotation of IWGCNA candidate genes.** Click here for file

Additional file 2 **Results for 89 genes that met the IWGCNA criteria out of the 8966 most varying genes.** Click here for file
