# Supplementary material for: Integrated Weighted Gene Co-expression Network Analysis with an Application to Chronic Fatigue Syndrome
Source: BMC Syst Biol. 2008 Nov 6;2:95. doi: 10.1186/1752-0509-2-95 (PMC2625353; doi:10.1186/1752-0509-2-95)
Supplement: Additional file 1 — Functional annotation of IWGCNA candidate genes. [file 1752-0509-2-95-S1.pdf]

Additional file 1. Functional annotations of 20 candidate genes from an IWGCNA of chronic fatigue syndrome data. All statistically significant genes were upregulated in the high severity group. 16 out of the 20 candidate genes were eligible for Ingenuity Pathways Analysis (IPA). (a) IPA identified four networks with a statistically significant interaction among 12 candidate genes (p-value ≈ 10^{-32}). Hematological disease was one of the three most significant functions for this interaction (other top functions can be found in the footnotes). (b) Analysis of the 212 network eligible blue module genes identified several significant pathways that have previously been shown to be involved in CFS. (c) Using a *LEO.NB.SingleMarker* threshold of 0.3 indicates that all but three of the candidate genes are causal for the blue module gene expression (where 66 total genes were causal by this criterion).

Gene Name and Genbank Accession	Full gene name. Entrez Gene and/or GeneRIFs description. Chromosome Location	Ingenuity Pathways Gene Annotation		c) Causality	
		a) 16 out of 20 Candidate Genes	b) 212 out of 299 Module Genes	LEO score[13]	Rank in Module
FOXN1 (NM_003593)	Forkhead box N1. Mutations result in a severely compromised immune system, T-cell immunodeficiency, skin disorder congenital alopecia. 17q11-q12	Hematological Disease[1] Rank = 1, p-value ≈ 10^{-32}	Cell Function[5] Rank = 8, p-value ≈ 10^{-16}	0.82	6
PRDX3 (AF118073)	Peroxiredoxin 3. Antioxidant function, regulates abundance of H_20_2, which promotes apoptosis. 10q25-q26	Hematological Disease[1]	Endocrine Disorders/ Inflammation[6] Rank = 6, p-value ≈ 10^{-20}	0.77	8
SUCLA2 (AK001458)	Succinate-CoA ligase, ADP-forming, beta subunit. Defects associated with encephalomyopathy. 13q12.2-q13.3	Hematological Disease[1]	Cell Cycle[7] Rank = 5, p-value ≈ 10^{-22}	0.77	9
TFB2M (AK026314)	Transcription factor B2, mitochondrial. 1q44	Hematological Disease[1]	Cell Cycle[7]	0.69	18
MED8 (BC010019)	Mediator complex subunit 8	Hematological Disease[1]	Amino Acid Met.[8] Rank = 1, p-value ≈ 10^{-45}	0.82	7
SNURF (AF101044)	SNRPN upstream reading frame. Alternative splicing/deletion leads to Angelman syndrome or Prader-Willi. 15q12	Hematological Disease[1]	Amino Acid Met.[8]	0.53	36
DCTN2 (NM_006400)	Dynactin 2 (p50). Required in peroxisome biogenesis. 12q13.2-q13.3	Hematological Disease[1]	Amino Acid Met.[8]	0.30	66
PGK1 (AB062432)	Phosphoglycerate kinase 1. Glycolysis. Xq13	Hematological Disease[1]	Amino Acid Met.[8]	-0.28	132
PRKCH (BC001000)	Protein kinase C, eta. Regulates keratinocyte differentiation. 14q22-q23	Hematological Disease[1]	Connective Tissue[9] Rank = 2, p-value ≈ 10^{-44}	-0.13	116

RYK (NM_002958)	RYK receptor-like tyrosine kinase. May play a role in the development of cleft lip and/or palate. 3q22	Hematological Disease[1]	Connective Tissue[9]	-0.50	182
VAMP5 (AF077197)	Vesicle-associated membrane protein 5 (myobrevin). Associated with myogenesis. 2p11.2	Hematological Disease[1]	Connective Tissue[10]<br>Rank = 7, p-value ≈ 10^{-18}	0.56	32
PBLD (AK027673)	Phenazine biosynthesis-like protein domain containing. 10pter-q25.3	Hematological Disease[1]	Connective Tissue[10]	0.41	50
NPAL2 (AK024017)	NIPA-like domain containing 2. 8q22.2	Digestive System[2]<br>Rank = 2, p-value ≈ 10^{-3}	Viral Function[11]<br>Rank = 3, p-value ≈ 10^{-32}	0.67	21
CD302 (BC020646)	C-type lectin receptor involved in cell adhesion and migration, as well as endocytosis and phagocytosis. 2q24.2	Carbohydrate Metabolism[3]<br>Rank = 2, p-value ≈ 10^{-3}	Viral Function[11]	0.38	55
PPP1R14C (AF407165)	Protein phosphatase 1, regulatory (inhibitor) subunit 14C. Enriched in brain, heart and skeletal muscle. 6q24.3-q25.3	Cancer[4]<br>Rank = 2, p-value ≈ 10^{-3}	Cell Proliferation[12]<br>Rank = 9, p-value ≈ 10^{-14}	0.83	5
TMEM50A (AF081282)	Transmembrane protein 50A. May contribute to RH haplotype selection. 1p36.11	NA, Rank = 2	NA, Rank = 14	0.39	53
CRNKL1 (AF111802)	Crooked neck pre-mRNA splicing factor-like 1. 20p11.2	NA	NA	0.77	10
LTV1 (AK027815)	Protein coding. 6q24.2	NA	NA	0.65	24
AF090939	Discontinued record.	NA	NA	0.85	3
XM13557	Unmapped.	NA	NA	0.57	28

[1] Cell Cycle, Cancer, Hematological Disease
[2] Digestive System D&F, Hepatic System D&F, Organ Dev.
[3] Carbohydrate Metabolism, Gene Expression, Genetic Disorder
[4] Cancer, Cellular Movement, Skeletal and Muscular Disorders
[5] Cell Fun. and Main., Small Molecule Biochem., Molecular Transport
[6] Endocrine System Disorders, Infectious Disease, Inflammatory Disease
[7] Cell Assembly and Org., Cell Cycle, DNA Replication/Recomb./Repair
[8] Post-Translational Modification, Amino Acid Metabolism, Molecular Transport
[9] Organ Morphology, Cell Morphology, Connective Tissue D&F
[10] Gene Expression, Cellular Development, Connective Tissue D&F
[11] Viral Function, Cell. Assembly and Org., Cell Fun. and Maintenance
[12] Post-Translational Modification, Cancer, Cellular Growth/Proliferation
[13] LEO.NB.SingleMarker scores (converted to fold changes).
