# Supplementary material for: Integrated Weighted Gene Co-expression Network Analysis with an Application to Chronic Fatigue Syndrome
Source: BMC Syst Biol. 2008 Nov 6;2:95. doi: 10.1186/1752-0509-2-95 (PMC2625353; doi:10.1186/1752-0509-2-95)
Supplement: Additional file 2 — Results for 89 genes that met the IWGCNA criteria out of the 8966 most varying genes. [file 1752-0509-2-95-S2.pdf]

Additional File 2. Gene names, accession numbers and Pearson correlations (r) for the 89 genes among the 8966 most varying genes that met the IWGCNA screening criteria (excluding module membership). The correlations were computed using all 127 samples studied (All), the 98 female samples and the 29 male samples except for the correlation with severity which only had 87 non-missing scores (64 female and 23 male). The CFS Severity column is bolded for clarity.

	**Pearson correlations with gene expression profiles**											
	MEblue		**CFS Severity**					*TPH2* SNP				
**Gene Name and GenBank**	r: All	Rank*	**r: All**	**p-value**	**q-value**	**r: M**	**r: F**	r: All	p-value	q-value	r: M	r: F
1 ACVR2B (NM_001106)	0.77	8338	**0.31**	**0.004**	**0.081**	**0.47**	**0.49**	0.22	0.011	0.885	0.38	0.21
2 AF090939	0.81	8656	**0.32**	**0.003**	**0.081**	**0.43**	**0.60**	0.21	0.016	0.885	0.25	0.24
3 AF130054	0.68	7325	**0.26**	**0.014**	**0.094**	**0.30**	**0.62**	0.11	0.210	0.885	0.25	0.27
4 AKR1B10 (AF052577)	0.79	8544	**0.27**	**0.011**	**0.091**	**0.40**	**0.44**	0.11	0.219	0.885	0.26	0.24
5 AL022341	0.78	8487	**0.31**	**0.003**	**0.081**	**0.36**	**0.51**	0.13	0.149	0.885	0.30	0.20
6 AL031055	0.75	8156	**0.27**	**0.012**	**0.092**	**0.35**	**0.40**	0.19	0.035	0.885	0.23	0.28
7 AP4S1 (AB030654)	0.76	8266	**0.26**	**0.015**	**0.097**	**0.50**	**0.44**	0.15	0.083	0.885	0.32	0.28
8 ARHGEF12 (AB002380)	0.79	8524	**0.32**	**0.003**	**0.081**	**0.53**	**0.47**	0.16	0.075	0.885	0.22	0.30
9 ATF3 (NM_004024)	0.58	5822	**0.16**	**0.138**	**0.218**	**0.27**	**0.39**	0.13	0.133	0.885	0.26	0.22
10 BAT3 (NM_004639)	0.60	6150	**0.22**	**0.041**	**0.134**	**0.33**	**0.38**	0.13	0.147	0.885	0.35	0.27
11 BC008826	0.66	7080	**0.22**	**0.038**	**0.131**	**0.36**	**0.42**	0.18	0.040	0.885	0.27	0.20
12 BCAS2 (AB020623)	0.55	5465	**0.28**	**0.008**	**0.085**	**0.38**	**0.47**	0.18	0.040	0.885	0.28	0.26
13 C11ORF9 (BC004938)	0.74	8022	**0.23**	**0.036**	**0.129**	**0.30**	**0.41**	0.17	0.056	0.885	0.26	0.29
14 C20ORF10 (AB017802)	0.73	7879	**0.23**	**0.029**	**0.121**	**0.26**	**0.44**	0.16	0.064	0.885	0.23	0.20
15 CCDC82 (AK027171)	0.81	8671	**0.22**	**0.040**	**0.133**	**0.29**	**0.49**	0.25	0.004	0.885	0.39	0.33
16 CD302 (BC020646)	0.62	6529	**0.24**	**0.028**	**0.117**	**0.28**	**0.42**	0.18	0.046	0.885	0.29	0.23
17 CDC23 (AB011472)	0.69	7481	**0.25**	**0.022**	**0.109**	**0.48**	**0.36**	0.16	0.065	0.885	0.37	0.21
18 CDH4 (AK091496)	0.85	8832	**0.31**	**0.003**	**0.081**	**0.39**	**0.55**	0.14	0.106	0.885	0.30	0.23
19 CDR2L (BC013105)	0.66	7057	**0.25**	**0.018**	**0.103**	**0.28**	**0.45**	0.18	0.044	0.885	0.21	0.24
20 CPSF6 (NM_007007)	0.72	7811	**0.28**	**0.009**	**0.087**	**0.43**	**0.47**	0.17	0.058	0.885	0.23	0.24
21 CRB1 (AY043325)	0.56	5482	**0.23**	**0.032**	**0.125**	**0.28**	**0.35**	0.22	0.014	0.885	0.37	0.26
22 CRNKL1 (AF111802)	0.75	8129	**0.27**	**0.012**	**0.092**	**0.41**	**0.47**	0.22	0.013	0.885	0.36	0.28
23 CXADR (BC003684)	0.69	7435	**0.22**	**0.042**	**0.134**	**0.31**	**0.42**	0.15	0.095	0.885	0.20	0.24
24 DCTN2 (NM_006400)	0.72	7785	**0.18**	**0.087**	**0.177**	**0.21**	**0.40**	0.23	0.009	0.885	0.41	0.20
25 DMBT1 (NM_004406)	0.77	8359	**0.30**	**0.005**	**0.081**	**0.50**	**0.41**	0.08	0.368	0.912	0.20	0.20
26 EDA (AF040628)	0.68	7359	**0.20**	**0.059**	**0.152**	**0.32**	**0.35**	0.15	0.093	0.885	0.22	0.20
27 EIF2C2 (AF121255)	0.76	8268	**0.30**	**0.005**	**0.081**	**0.29**	**0.41**	0.18	0.044	0.885	0.27	0.24
28 F11 (AF045649)	0.75	8109	**0.27**	**0.011**	**0.091**	**0.22**	**0.43**	0.16	0.068	0.885	0.26	0.23
29 FBXL17 (XM_098421)	0.79	8501	**0.28**	**0.009**	**0.087**	**0.43**	**0.40**	0.13	0.137	0.885	0.24	0.20

30	FIP1L1 (NM_030917)	0.82	8694	**0.25**	**0.020**	**0.106**	**0.26**	**0.45**	0.17	0.060	0.885	0.26	0.20
31	FOXN1 (NM_003593)	0.75	8136	**0.21**	**0.055**	**0.147**	**0.27**	**0.44**	0.21	0.018	0.885	0.23	0.24
32	GART (NM_000819)	0.62	6392	**0.29**	**0.007**	**0.084**	**0.28**	**0.42**	0.09	0.309	0.892	0.20	0.36
33	GLI1 (AF316573)	0.46	4061	**0.26**	**0.014**	**0.094**	**0.24**	**0.43**	0.22	0.013	0.885	0.27	0.31
34	HSP90AA2 (M30627)	0.80	8566	**0.29**	**0.006**	**0.083**	**0.33**	**0.50**	0.11	0.222	0.885	0.26	0.28
35	IKBKB (BC006231)	0.79	8521	**0.26**	**0.016**	**0.099**	**0.31**	**0.39**	0.13	0.139	0.885	0.28	0.22
36	IL6ST (AB015706)	0.75	8186	**0.36**	**0.001**	**0.081**	**0.39**	**0.54**	0.15	0.090	0.885	0.24	0.23
37	INOC1 (AK002176)	0.78	8453	**0.22**	**0.045**	**0.137**	**0.27**	**0.37**	0.13	0.133	0.885	0.21	0.23
38	LIMA1 (AF157325)	0.75	8154	**0.35**	**0.001**	**0.081**	**0.30**	**0.56**	0.18	0.040	0.885	0.27	0.21
39	LTV1 (AK027815)	0.81	8626	**0.30**	**0.005**	**0.081**	**0.39**	**0.49**	0.19	0.029	0.885	0.29	0.21
40	MED8 (BC010019)	0.85	8862	**0.29**	**0.007**	**0.085**	**0.31**	**0.52**	0.22	0.015	0.885	0.35	0.29
41	MYLK (NM_053026)	0.74	8078	**0.22**	**0.037**	**0.130**	**0.47**	**0.38**	0.15	0.090	0.885	0.35	0.24
42	NCR1 (AJ006122)	0.71	7656	**0.28**	**0.009**	**0.087**	**0.37**	**0.46**	0.15	0.082	0.885	0.25	0.31
43	NDFIP1 (AK075524)	0.76	8238	**0.24**	**0.028**	**0.118**	**0.24**	**0.41**	0.25	0.005	0.885	0.21	0.26
44	NKAP (AK026279)	0.76	8300	**0.25**	**0.022**	**0.109**	**0.36**	**0.44**	0.23	0.008	0.885	0.28	0.24
45	NPAL2 (AK024017)	0.90	8954	**0.35**	**0.001**	**0.081**	**0.37**	**0.61**	0.21	0.020	0.885	0.33	0.22
46	NUP37 (AK026271)	0.70	7512	**0.27**	**0.013**	**0.093**	**0.34**	**0.47**	0.14	0.130	0.885	0.28	0.21
47	PBLD (AK027673)	0.84	8777	**0.31**	**0.003**	**0.081**	**0.28**	**0.60**	0.17	0.049	0.885	0.23	0.21
48	PCM1 (AK091406)	0.07	595	**0.25**	**0.020**	**0.105**	**0.32**	**0.44**	-0.07	0.420	0.924	0.29	0.24
49	PDPK1 (BC006339)	0.85	8853	**0.35**	**0.001**	**0.081**	**0.47**	**0.54**	0.16	0.078	0.885	0.24	0.24
50	PGK1 (AB062432)	0.74	8072	**0.22**	**0.045**	**0.137**	**0.37**	**0.43**	0.14	0.108	0.885	0.26	0.21
51	PIF1 (AK026345)	0.85	8843	**0.32**	**0.003**	**0.081**	**0.24**	**0.58**	0.10	0.282	0.886	0.20	0.24
52	PPP1R14C (AF407165)	0.85	8859	**0.24**	**0.025**	**0.113**	**0.21**	**0.49**	0.21	0.016	0.885	0.26	0.24
53	PRDX3 (AF118073)	0.82	8711	**0.26**	**0.017**	**0.100**	**0.43**	**0.44**	0.21	0.020	0.885	0.32	0.23
54	PRKCH (BC001000)	0.68	7295	**0.16**	**0.143**	**0.220**	**0.27**	**0.38**	0.15	0.089	0.885	0.23	0.20
55	PSMB8 (U17497)	0.58	5866	**0.19**	**0.071**	**0.164**	**0.22**	**0.39**	0.23	0.009	0.885	0.37	0.30
56	PTPN2 (BC008244)	0.72	7864	**0.30**	**0.005**	**0.081**	**0.55**	**0.48**	0.25	0.005	0.885	0.25	0.25
57	RAB3B (AF498932)	0.81	8636	**0.34**	**0.001**	**0.081**	**0.49**	**0.57**	0.18	0.040	0.885	0.34	0.24
58	RNFT2 (BC011878)	0.81	8677	**0.35**	**0.001**	**0.081**	**0.42**	**0.65**	0.17	0.058	0.885	0.40	0.22
59	RYK (NM_002958)	0.72	7801	**0.21**	**0.048**	**0.140**	**0.26**	**0.44**	0.12	0.182	0.885	0.21	0.21
60	SEC23B (BC005032)	0.70	7635	**0.32**	**0.002**	**0.081**	**0.39**	**0.50**	0.13	0.133	0.885	0.25	0.21
61	SLC7A1 (AF078107)	0.71	7650	**0.18**	**0.099**	**0.186**	**0.28**	**0.36**	0.22	0.013	0.885	0.40	0.24
62	SNURF (AF101044)	0.80	8568	**0.27**	**0.012**	**0.092**	**0.51**	**0.46**	0.18	0.037	0.885	0.32	0.23
63	SNX15 (NM_013306)	0.88	8934	**0.35**	**0.001**	**0.081**	**0.44**	**0.59**	0.20	0.025	0.885	0.30	0.24
64	SUCLA2 (AK001458)	0.67	7219	**0.20**	**0.059**	**0.151**	**0.25**	**0.38**	0.20	0.021	0.885	0.36	0.22
65	TAS2R16 (AF227139)	0.60	6209	**0.20**	**0.058**	**0.150**	**0.22**	**0.46**	0.13	0.158	0.885	0.22	0.21
66	TCEAL7 (BC016786)	0.86	8892	**0.37**	**0.000**	**0.081**	**0.50**	**0.63**	0.16	0.069	0.885	0.31	0.28

67	TFB2M (AK026314)	0.86	8877	**0.31**	**0.004**	**0.081**	**0.29**	**0.57**	0.21	0.016	0.885	0.22	0.22
68	TH1L (AF161479)	0.81	8674	**0.29**	**0.006**	**0.083**	**0.40**	**0.49**	0.15	0.098	0.885	0.32	0.26
69	TMEM111 (AF157321)	0.78	8429	**0.23**	**0.033**	**0.125**	**0.26**	**0.41**	0.16	0.067	0.885	0.27	0.20
70	TMEM134 (AK025402)	0.71	7728	**0.25**	**0.019**	**0.105**	**0.34**	**0.41**	0.16	0.066	0.885	0.33	0.22
71	TMEM50A (AF081282)	0.79	8545	**0.26**	**0.014**	**0.094**	**0.29**	**0.47**	0.17	0.050	0.885	0.22	0.26
72	TP53INP2 (BC035639)	0.68	7376	**0.12**	**0.256**	**0.287**	**0.21**	**0.37**	0.14	0.114	0.885	0.34	0.20
73	TPD52L2 (AF004429)	0.84	8802	**0.29**	**0.006**	**0.083**	**0.39**	**0.49**	0.17	0.052	0.885	0.32	0.25
74	TRIM45 (BC034943)	0.85	8849	**0.31**	**0.003**	**0.081**	**0.40**	**0.52**	0.16	0.075	0.885	0.26	0.29
75	TRIM7 (BC011567)	0.68	7347	**0.31**	**0.004**	**0.081**	**0.32**	**0.51**	0.17	0.056	0.885	0.28	0.23
76	TRPA1 (NM_007332)	0.62	6498	**0.16**	**0.132**	**0.213**	**0.24**	**0.38**	0.10	0.272	0.885	0.22	0.27
77	TXNDC12 (AF131758)	0.84	8815	**0.33**	**0.002**	**0.081**	**0.31**	**0.67**	0.17	0.051	0.885	0.21	0.22
78	UBQLN1 (NM_013438)	0.75	8168	**0.17**	**0.122**	**0.204**	**0.25**	**0.41**	0.17	0.055	0.885	0.31	0.28
79	USP34 (NM_014709)	0.91	8962	**0.32**	**0.002**	**0.081**	**0.48**	**0.53**	0.16	0.071	0.885	0.25	0.23
80	VAMP5 (AF077197)	0.85	8826	**0.33**	**0.002**	**0.081**	**0.47**	**0.60**	0.24	0.007	0.885	0.35	0.27
81	XM_055874	0.82	8692	**0.34**	**0.001**	**0.081**	**0.40**	**0.56**	0.19	0.031	0.885	0.26	0.23
82	XM_067644	0.81	8632	**0.29**	**0.007**	**0.083**	**0.30**	**0.57**	0.27	0.002	0.885	0.35	0.29
83	XM_083941	0.36	2826	**0.27**	**0.013**	**0.093**	**0.32**	**0.36**	0.18	0.041	0.885	0.24	0.23
84	XM_086132	0.74	8061	**0.23**	**0.036**	**0.129**	**0.32**	**0.44**	0.26	0.003	0.885	0.36	0.35
85	XM13557	0.81	8628	**0.32**	**0.002**	**0.081**	**0.36**	**0.52**	0.19	0.032	0.885	0.38	0.23
86	XM66127	0.85	8821	**0.26**	**0.014**	**0.095**	**0.45**	**0.43**	0.18	0.040	0.885	0.24	0.25
87	XM73039	0.63	6589	**0.29**	**0.006**	**0.083**	**0.36**	**0.53**	0.17	0.055	0.885	0.36	0.20
88	ZAK (AB049734)	0.83	8763	**0.24**	**0.026**	**0.114**	**0.45**	**0.45**	0.18	0.040	0.885	0.22	0.22
89	ZMYND11 (NM_006624)	0.79	8506	**0.31**	**0.004**	**0.081**	**0.47**	**0.48**	0.13	0.155	0.885	0.32	0.21

*The rank of each gene in terms of its MEblue correlation out of the 8966 genes used to start the analysis.
